# Disruption of structural connectome hierarchy in age-related hearing loss

**DOI:** 10.3389/fnins.2025.1555553

**Published:** 2025-03-17

**Authors:** Yi Zhen, Hongwei Zheng, Yi Zheng, Zhiming Zheng, Yaqian Yang, Shaoting Tang

**Affiliations:** ^1^School of Mathematical Sciences, Beihang University, Beijing, China; ^2^Key Laboratory of Mathematics, Informatics and Behavioral Semantics, Beihang University, Beijing, China; ^3^Beijing Academy of Blockchain and Edge Computing, Beijing, China; ^4^Institute of Artificial Intelligence, Beihang University, Beijing, China; ^5^Hangzhou International Innovation Institute, Beihang University, Hangzhou, China; ^6^Institute of Medical Artificial Intelligence, Binzhou Medical University, Yantai, China; ^7^Zhongguancun Laboratory, Beijing, China; ^8^Beijing Advanced Innovation Center for Future Blockchain and Privacy Computing, Beihang University, Beijing, China; ^9^State Key Laboratory of Complex & Critical Software Environment, Beihang University, Beijing, China

**Keywords:** hierarchical organization, structural gradient, age-related hearing loss, transcriptional signatures, brain network, diffusion magnetic resonance imaging

## Abstract

**Introduction:**

Age-related hearing loss (ARHL) is a common sensory disability among older adults and is considered a risk factor for the development of dementia. Previous work has shown altered brain connectome topology in ARHL, including abnormal nodal strength and clustering coefficient. However, whether ARHL affects the hierarchical organization of structural connectome and how these alterations relate to transcriptomic signatures remain unknown.

**Methods:**

Here, we apply a gradient mapping framework to the structural connectome derived from diffusion magnetic resonance imaging. We focus on the first three structural gradients that reflect distinct hierarchical organization of structural connectome, and assess ARHL-related changes.

**Results:**

We find that, compared to controls, ARHL patients exhibit widespread disruptions of structural connectome organization, spanning from primary sensory areas (e.g., somatomotor network) to high-order association areas (e.g., default mode network). Subsequently, by employing subcortical-weighted gradients derived from weighting cortical gradients by subcortical-cortical connectivity, we observe that ARHL patients show significantly altered subcortical-cortical connectivity in the left caudate, left nucleus accumbens, right hippocampus, and right amygdala. Finally, we investigate the relationship between gene expression and alterations in structural gradients. We observe that these alterations in structural gradients are associated with weighted gene expression profiles, with relevant genes preferentially enriched for inorganic ion transmembrane transport and terms related to regulating biological processes.

**Discussion:**

Taken together, these findings highlight that ARHL is associated with abnormal structural connectome hierarchy and reveal the transcriptomic relevance of these abnormalities, contributing to a richer understanding of the neurobiological substrates in ARHL.

## 1 Introduction

Age-related hearing loss (ARHL) is a prevalent sensory impairment that affects more than 40% of adults over 50 years old, resulting in social isolation, communication difficulties, and diminished quality of life (Eckert et al., 2012; Slade et al., 2020). Individuals with ARHL are considered to have an increased risk of cognitive deficits and dementia (Ford et al., 2018; Liu and Lee, 2019; Slade et al., 2020). Previous neuroimaging studies have demonstrated that ARHL patients exhibit disrupted brain networks in both local and global characteristics (Guan et al., 2022; Ponticorvo et al., 2022; Xu et al., 2024). For instance, compared with healthy controls, ARHL patients showed a significant increase in global efficiency and clustering coefficient of functional networks (Guan et al., 2022; Ponticorvo et al., 2022), as well as changes in local nodal strength of structural networks in several regions (Ponticorvo et al., 2022). Through the lens of brain connectome analysis and graph theory, these studies provide valuable insights into the neuropathological mechanisms underlying ARHL.

By compressing macroscale brain connectomics into a low-dimensional embedding space, the recently developed gradient mapping technique offers an appealing framework to elucidate systematic organizational principles of brain connectome (Margulies et al., 2016; Hong et al., 2019; Park et al., 2021b). Different from graph theory that characterizes local and global topological properties of networks, this technique generates a series of spatial arrangements (called gradients) that capture continuous variations in connectivity profiles, with tightly interconnected regions positioned proximally along gradient axes. The spatial variation of gradient informs how connectivity profiles of distributed regions are integrated and segregated (Huntenburg et al., 2018; Bayrak et al., 2019). For example, the principal gradient derived from resting-state functional connectivity represents a macroscale organization that differentiates between transmodal default mode areas and unimodal sensory areas (Margulies et al., 2016). A substantial body of research has utilized gradient mapping to characterize alterations in organization features of functional and structural connectivity during development and aging (Bethlehem et al., 2020; Park et al., 2021a; Dong et al., 2021), as well as neuropsychiatric disorders (Hong et al., 2019; Park et al., 2021b; Xia et al., 2022). Specially, one recent study reported that ARHL patients exhibited alterations in the principal gradient of functional connectivity in the visual and default mode networks, suggesting abnormal functional organization (Tong et al., 2023). Nevertheless, it remains unclear whether hierarchical organizations of structural connectivity are similarly altered in ARHL patients. In addition, preliminary evidence indicates that ARHL is associated with changes in subcortical structures (Xu et al., 2019; Chen et al., 2020), yet there are limited studies examining subcortical-cortical structural connectivity in ARHL patients. It remains uncertain whether alterations in structural gradients are accompanied with subcortical impairments. Given the potential relationship between functional and structural connectomes (Yang et al., 2023), identifying altered structural connectome hierarchy provides valuable insights into functional network abnormalities observed in ARHL.

The availability of spatially comprehensive whole-brain transcriptomic maps, such as the Allen Human Brain Atlas (AHBA) (Hawrylycz et al., 2012), has provided opportunities to assess relations between gene expression and neuroimaging phenotypes. Prior studies have explored the spatial correspondence between gene expression profiles and regional variations in neuroimaging phenotypes (Morgan et al., 2019; Li et al., 2021), giving insights into potential molecular substrates that underlie altered phenotypes. For example, abnormalities in functional or structural connectome organization have been associated with gene expression patterns in multiple brain disorders, including autism (Park et al., 2021b), depression (Xiao et al., 2023), and Alzheimer's disease (Zheng et al., 2024). However, whether and how alterations in structural connectome organization in ARHL relate to gene expression remains unclear.

Here, we sought to investigate whether hierarchical organizations of structural connectome are altered in ARHL patients, and if so, provide further insights into the potential molecular mechanisms underlying these changes. To achieve this, we utilized the diffusion mapping method to estimate the first three structural gradients in both controls and patients. We hypothesized that ARHL patients would show significantly different structural gradients compared to controls. Subsequently, we employed subcortical-weighted gradients to examine whether ARHL patients were associated with abnormal subcortical-cortical structural connectivity. Finally, we applied a partial least squares (PLS) regression to investigate the relationship between alterations in structural gradients and transcriptomic data.

## 2 Materials and methods

### 2.1 Participants and data acquisition

The dataset is obtained from the Hearing loss Connectome (ds005026) (Ponticorvo et al., 2022) that is available on the OpenNeuro platform (Markiewicz et al., 2021). Fifty-two ARHL patients (16 female; 63.67 ± 7.80 years old) and thirty normal hearing controls (20 female; 59.53 ± 7.17 years old) were included. All participants had no history of neurological and/or psychiatric illness, ear surgery, or specific contraindications to magnetic resonance. Pure-tone audiometry and speech audiometry were conducted to evaluate participants' audiological status. More details on auditory evaluation can be found in Ponticorvo et al. (2022). All participants provided written informed consent, and experimental procedures were approved by the institutional review board of the University of Salerno.

The MRI data were collected on a 3T Siemens Skyra scanner with a 20-channel RF head-and-neck coil. The T1-weighted images were acquired with an MPRAGE sequence using the following parameters: TR = 2.4 s, TE = 2.26 ms, TI = 0.95 s, flip angle = 8°, matrix = 256 × 256, voxel size = 1 × 1 × 1. The diffusion-weighted images were collected with a multi-band accelerated echo-planar sequence using the following parameters: TR = 4.71 s, TE = 0.0906 s, Acceleration Factor = 2, flip angle = 90°, voxel size = 2 × 2 × 2, 1 volume with b = 0 s/mm^2^, 64 noncollinear directions with b = 1500 s/mm^2^. A diffusion-weighted scan with opposite phase encoding directions was acquired to correct susceptibility distortions.

### 2.2 Data preprocessing

All T1-weighted images were subjected to tissue segmentation and cortical surface reconstruction by FreeSurfer's recon-all pipeline (version: 7.4.1) (Dale et al., 1999; Fischl, 2012). Diffusion-weighted data were processed using FSL (version: 6.0.7) (Jenkinson et al., 2012), MRtrix3 (version: 3.0.4) (Tournier et al., 2019), and MRtrix3Tissue (version: 5.2.9, https://3Tissue.github.io). The preprocessing procedures included denoising (Veraart et al., 2016; Cordero-Grande et al., 2019), correction for susceptibility distortions (Andersson et al., 2003), corrections of motion and eddy current distortions (Andersson and Sotiropoulos, 2016; Bastiani et al., 2019; Andersson et al., 2016), bias field correction (Tustison et al., 2010), and estimation of brain mask (Hoopes et al., 2022). Individual structural connectome was derived from the preprocessed diffusion data. We estimated the response functions of different tissues using the Dhollander algorithm (Dhollander et al., 2019). Fiber orientation distributions were reconstructed using the single-shell 3-tissue constrained spherical deconvolution method (Dhollander and Connelly, 2016) and were intensity normalized in the log-domain (Raffelt et al., 2017; Dhollander et al., 2021). A whole-brain tractography with 5 million streamlines was generated using a probabilistic approach (iFOD2) (Tournier et al., 2010) and anatomically constrained tractography (ACT) algorithm (Smith et al., 2012, 2020) with dynamic seeding, and the estimation of tract weights (SIFT2) (Smith et al., 2015) to reduce reconstruction biases. The Schaefer-400 parcellation (Schaefer et al., 2018) was mapped onto the individual diffusion-weighted space to create cortical structural connectivity. Eight bilateral subcortical structures (including the thalamus, caudate, putamen, pallidum, hippocampus, amygdala, accumbens, and ventral diencephalon) derived from the FreeSurfer's segmentation (Dale et al., 1999) were used to construct subcortical-cortical connectivity. The structural connectivity between pairs of regions was further scaled by the inverse of two region volumes (Hagmann et al., 2008). Quality control of T1-weighted images was done by visual inspection, and two participants with excessive head movement or poor cortical segmentation were excluded. Diffusion data from two participants with high total outliers were excluded. Additionally, one participant's diffusion data had a different phase encoding direction from other participants and was also excluded from this study. Finally, 77 participants (49 patients) were retained for the subsequent analysis.

### 2.3 Structural connectome gradients

For each participant, we estimated structural connectome gradients using the BrainSpace toolbox (Vos de Wael et al., 2020). Specifically, we constructed an affinity matrix by calculating the cosine similarity between regional structural connectivity profiles (Park et al., 2021b). Due to the sparsity of structural connectome, this step was implemented on individual structural connectome that was not thresholded (Kim et al., 2024). We then performed a nonlinear diffusion map embedding (Coifman et al., 2005) of the affinity matrix to obtain multiple continuous components (i.e., structural gradients) that were arranged in descending order of explained variance. The procedure treats the affinity matrix as a graph and estimates the low-dimensional embedding from the high-dimensional connectivity matrix. Along low-dimensional axes, regions that are tightly interconnected are closer together, while regions with weak interconnection are farther apart (Huntenburg et al., 2018). Diffusion map embedding was affected by two parameters t and α. Consistent with previous studies (Margulies et al., 2016; Park et al., 2021a), we set t = 0 and α = 0.5 to preserve global relationships between points in the embedded space. To ensure the comparability between participants' structural gradients, we constructed a group-level gradient template. In accordance with prior studies (Zarkali et al., 2021; Yang et al., 2024), we averaged all structural connectome matrices from both patients and controls to generate the group-level structural connectome. We estimated the group-level gradient template from the group-level structural connectome and aligned the structural gradients of each participant to the template via Procrustes alignment (Langs et al., 2015). Procrustes alignment has been extensively utilized to rotate individual-level gradients to achieve maximum similarity with the template gradients, without applying a scaling factor (Hong et al., 2019; Xia et al., 2022; Vos de Wael et al., 2020). Procrustes alignment determines an optimal linear transformation S between the unaligned gradients G and the template gradients M, which minimizes the sum of squared errors between the aligned gradients (G*S) and template gradients M. In other words, the aligned gradient is obtained through a linear combination of the unaligned gradients.

### 2.4 Between-group differences in structural gradients

In agreement with previous work (Park et al., 2021b; Yoo et al., 2024), we applied multivariate analyses to compare differences in the first three structural gradients between ARHL patients and controls. In multivariate analyses, we employed Hotelling's T to identify the shared effects of ARHL across the three structural gradients. We conducted between-group comparisons at both network-level and region-level. To be specific, for network-level analyses, we averaged regional gradient scores according to Yeo's seven functional systems (Yeo et al., 2011), which included the visual, somatomotor, dorsal attention, ventral attention, limbic, frontoparietal, and default mode networks. We used multivariate analyses to compare network-level differences across the first three structural gradients, with statistical significance set at FDR-corrected p < 0.05. We then performed single-gradient comparisons on each gradient separately, using the univariate linear model. Multiple comparisons were corrected by the FDR method (corrected p < 0.05). For region-level analyses, we conducted multivariate analyses to assess between-group differences in gradient scores of each region across the first three structural gradients, with statistical significance set at FDR-corrected p < 0.05. We then performed post-hoc analyses to examine the contributions of each gradient to the overall effects, while correcting for the number of considered structural gradients (p < 0.05/3) (Wan et al., 2023; Yang et al., 2024). We also conducted regional comparisons on each gradient using the univariate linear model, with statistical significance set at FDR-corrected p < 0.05. In all comparisons, age and gender were included as covariates. All multivariate analyses were performed using the BrainStat toolbox (Larivière et al., 2023). Surface visualizations of between-group differences in structural gradients were generated using the Python packages BrainSpace (Vos de Wael et al., 2020) and Surfplot (Gale et al., 2021).

### 2.5 Meta-analysis

To understand the cognitive implication of brain regions with significant ARHL-related alterations, we performed a meta-analytic function decoding using the Python package NiMARE (Salo et al., 2022). We only retained significant cortical regions to get a thresholded Hotelling's T map. The decoding process correlated the thresholded map with all meta-analytic maps in the NeuroSynth database (Yarkoni et al., 2011). We only retained the top 15 terms relevant to cognitive behaviors or functions.

### 2.6 Subcortical-weighted gradients

We capitalized on the subcortical-weighted gradient to assess ARHL-related alterations in subcortical-cortical connectivity. Consistent with previous studies (Park et al., 2021b; Lee et al., 2023; Xiao et al., 2023), the subcortical-weighted gradient for each subcortical region was generated through element-wise multiplication between the structural gradient and the subcortical-cortical structural connectivity. We then averaged each subcortical-weighted gradient to extract nodal degree values. Multivariate analyses were implemented to compare between-group differences in nodal degree values along the first three gradients, with age and gender as covariates. We then used the univariate linear model to evaluate differences in degree values corresponding to each subcortical-weighted gradient. Multiple comparisons were adjusted by the FDR method (corrected p < 0.05). Subcortical visualizations of between-group differences in subcortical-weighted gradients were based on the R packages ggplot2 (Wickham, 2011) and ggseg (Mowinckel and Vidal-Piñeiro, 2020).

### 2.7 Transcriptomic-neuroimaging association analysis

We utilized transcriptomic data from the Allen Human Brain Atlas (AHBA) database (Hawrylycz et al., 2012) to examine the relationship between between-group differences in structural gradients and gene expression profiles. The AHBA database had postmortem microarray data in 3,702 different brain tissue samples from six neurotypical donors (1 female and 5 males, aged from 24 to 57 years). Given that these postmortem microarray data from the AHBA database are the only publicly available high spatial resolution gene expression atlas, it is a compromise choice to analyze the transcriptional association of ARHL-related structural alterations. Notably, although these data are derived from healthy donors, prior studies have utilized these data to explore transcriptional signatures of structural alterations in neuropsychiatric disorders (Morgan et al., 2019; Park et al., 2021b) and age-related neurodegenerative diseases (Thomas et al., 2021; Estevez-Fraga et al., 2023). Particularly, a recent study has employed these data to investigate the relationship between gene expression and the association between hearing ability in older adults and cortical morphology (Qiu et al., 2024). Given that only two donors contained gene expression data of the right hemisphere, we restricted the analysis to the left hemisphere. We preprocessed the microarray data using the Python package abagen (Markello et al., 2021; Arnatkeviciute et al., 2019), which included (1) updating the MNI coordinates of all tissue samples, (2) reannotating microarray probe-to-gene mappings, (3) intensity-based filtering of probes, (4) probe selection based on the highest differential stability in donors from the probes, (5) mapping tissue samples to regions defined by the Schaefer-400 parcellation, (6) normalizing expression data using a scaled robust sigmoid function, (7) calculating regional expression values. When none of the donors have assigned a tissue sample to a region in the functional parcellation, the expression value of the tissue sample closest to the centroid of the region will be assigned to that region. The process eventually yielded expression values of 15, 633 genes in 200 regions.

We used a partial least squares (PLS) regression to assess the association between group differences in gradients and gene transcription profiles. The PLS regression extracted components that were related to group differences in gradients from transcription profiles of 15, 633 genes. Consistent with prior work (Morgan et al., 2019; Li et al., 2021), the statistical significance of the variance explained by PLS components was assessed by 10, 000 permutation tests. We calculated the spatial correlation between PLS scores and case-control differences in structural gradients and evaluated its statistical significance via 10,000 spin permutation tests. The variability of PLS weight was estimated using 2,000 bootstrap resamples of 200 brain regions. The Z score of PLS weight was evaluated as the weight divided by its bootstrap standard deviation. According to the Z scores of PLS weights, we chose significant genes (one-sample Z tests, corrected *p* < 0.05) for subsequent analysis. Based on the sign of PLS weights, the selected genes were categorized into two sets (PLS+ and PLS- gene sets).

To understand the functional implications of the selected genes, we performed functional enrichment analysis for the PLS+ and PLS- gene sets, respectively, using the online software Metascape (Zhou et al., 2019). The enrichment categories included Gene Ontology (GO) Biological Process, KEGG Pathway, WikiPathways, Canonical Pathways, and Reactome Gene Sets. The results of the enrichment analysis were adjusted by the FDR method, and the significance threshold was set to corrected *p* < 0.05.

## 3 Results

### 3.1 Structural connectome gradients

**Figure 1:**
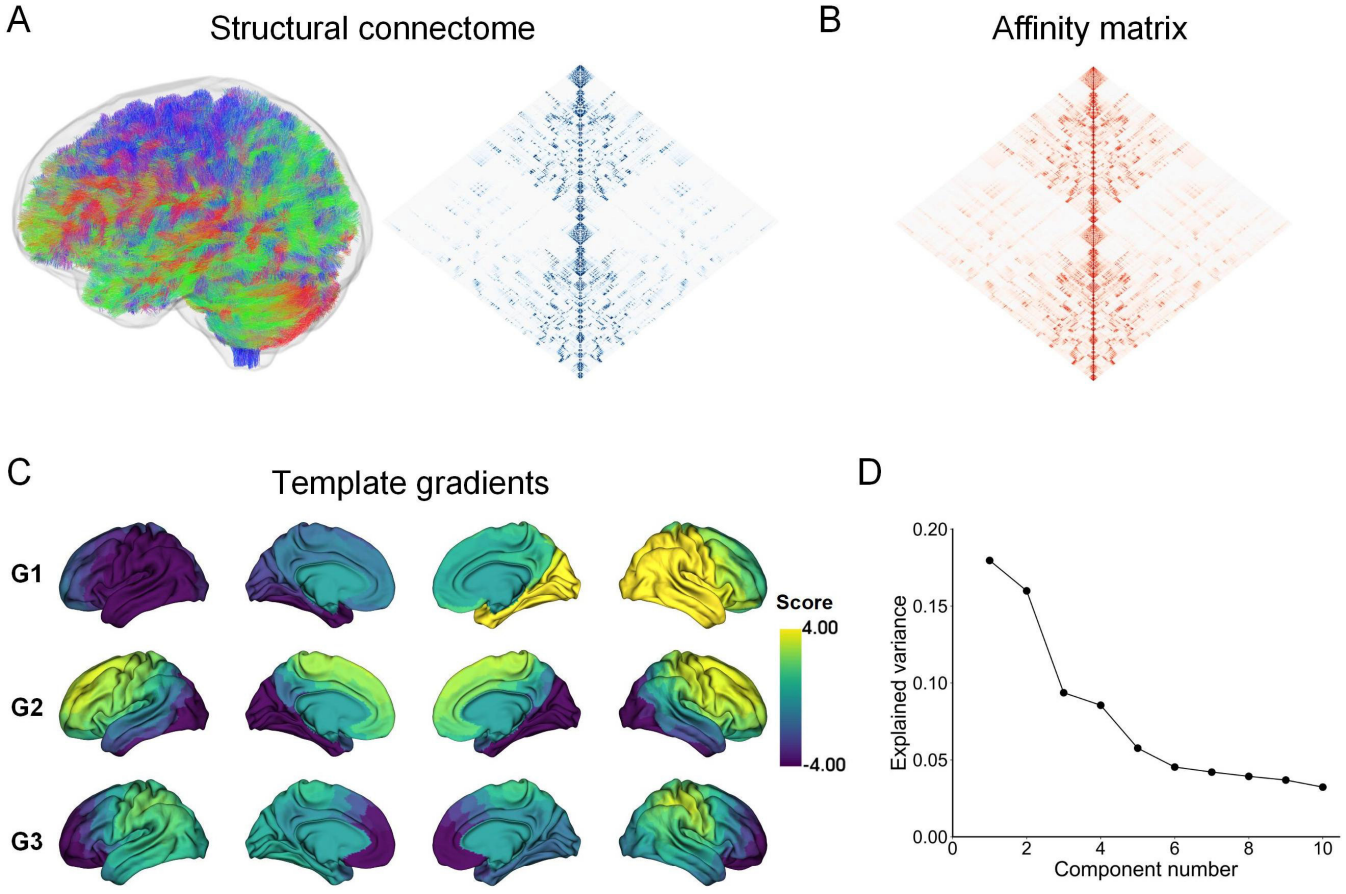
Pipeline for estimating structural connectome gradients. **(A)** The structural connectome is constructed using whole-brain probabilistic tractography. **(B)** The affinity matrix is derived by calculating the cosine similarity between regional structural connectome profiles. **(C)** The first three structural gradient templates are constructed by applying the diffusion map embedding algorithm to the group-level affinity matrix. **(D)** Variance accounted for by the first ten gradient components.

Through the diffusion map embedding algorithm, we generated the first three structural gradient templates (G1, G2, and G3) (Figures 1A–C). The three gradients accounted for approximately 43.33% of the total variance in the template connectome (Figure 1D). Consistent with previous literature (Park et al., 2021b; Noh et al., 2024), the first gradient (G1) captures a left-to-right hierarchical organization. The second gradient (G2) reflects a hierarchy traversing from anterior to posterior. The third gradient (G3) delineates a hierarchy axis where the prefrontal and lateral parietal/motor regions are situated at opposite ends (Figure 1C). To ensure direct comparability of structural gradients across individuals and groups, we aligned individual structural gradients to the template gradients using Procrustes alignment. The first three structural gradients of the group averages for the control and ARHL groups were presented in Supplementary Figure S1. Visually inspected, their spatial patterns are highly similar to the template gradients.

### 3.2 Network-level analysis

**Figure 2:**
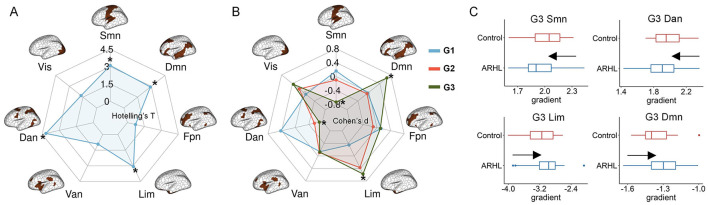
Network-level comparisons of structural gradients between controls and ARHL patients. **(A)** The multivariate analyses using Hotelling‘s T to identify shared effects across the first three structural gradients. *denotes significant group differences. **(B)** Network-level ARHL-control differences in a single gradient. *denotes significant group differences. **(C)** The significant ARHL-control differences in structural gradients in single-gradient comparisons. In each boxplot, the central line of the box indicates the median, the top/bottom edge of the box denotes the 75th/25th percentiles, and the whiskers of the box indicate the upper/lower bounds of 1.5 × the interquartile range. Resting-state networks: Vis, visual; Smn, somatomotor; Dan, dorsal attention; Van, ventral attention; Lim, limbic; Fpn, frontoparietal; Dmn, default mode.

In this section, we examined whether ARHL altered structural gradients, and if so, whether these alterations were concentrated in specific functional systems. We performed multivariate analyses using Hotelling's T to investigate shared effects across the first three gradients. Network-level analyses showed that, compared with controls, ARHL patients exhibited significant alterations in structural connectome organization across multiple networks, including the somatomotor, dorsal attention, limbic, and default mode networks (Figure 2A). We further conducted case-control comparisons in a single gradient. We detected no significant differences in G1 and G2 (Figure 2B). For G3, we observed significantly reduced gradient scores in the somatomotor and dorsal attention networks, and significantly increased gradient scores in the limbic and default mode networks in ARHL patients (Figures 2B, C). The detailed results of network-level analyses were reported in Supplementary Table S1.

### 3.3 Region-level analysis

**Figure 3:**
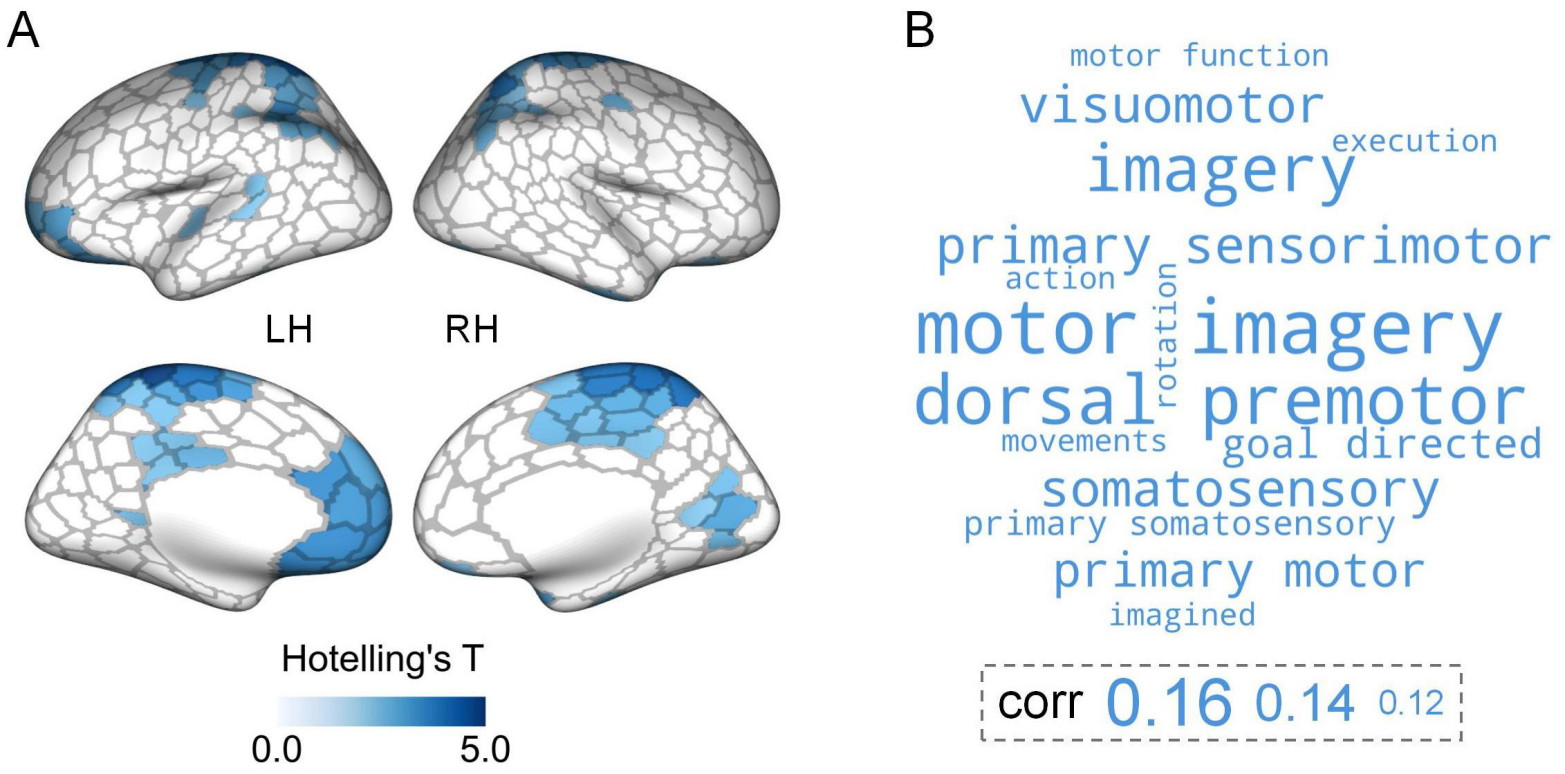
Region-level comparisons of structural gradients between controls and ARHL patients. **(A)** Significant ARHL-control differences in the first three structural gradients identified by multivariate analyses. The brain maps are colored according to Hotelling's T-values (FDR-corrected *p* < 0.05). LH, left hemisphere; RH, right hemisphere. **(B)** The top 15 cognitive terms from meta-analysis. The corr values represent the correlations between the thresholded Hotelling's T map and meta-analytic maps. The larger font size of a term indicates a stronger correlation between the thresholded Hotelling's T map and the meta-analytic map corresponding to that term.

We subsequently explored the between-group differences in hierarchical organization across the first three gradients at the regional level. Multivariate analyses revealed significant group differences in structural gradients in multiple cortical areas. These regions were predominantly situated in the lateral parietal cortex, motor cortex, paracentral lobule, lateral temporal cortex, cingulate, right visual areas, and prefrontal cortex (especially orbitofrontal cortex) (Figure 3A). Functional decoding analysis suggested that these significant regions were primarily related to motor-related functions (Figure 3B, Supplementary Table S2). For single-gradient comparisons, post-hoc analyses indicated that group differences in structural gradients were present in G2 and G3 (Supplementary Figure S2). Specifically, for G2, ARHL patients showed significantly higher gradient scores in two regions located in the left cingulate and right temporal pole respectively. In contrast, ARHL patients exhibited lower gradient scores in one region in the right superior parietal cortex. For G3, lower gradient scores were observed in the bilateral precentral, postcentral, paracentral, and superior parietal regions in ARHL patients. Conversely, higher gradient scores were in the left medial prefrontal and right visual regions in ARHL patients.

We performed regional comparisons at the single-gradient level using the univariate linear model. For G1 and G2, no significant between-group differences in gradient scores were detected. For G3, we found that ARHL patients showed significantly reduced gradient scores in 15 brain parcels, primarily located in the superior parietal lobule, precentral gyrus, postcentral gyrus, and paracentral lobule (Supplementary Figure S3A). We observed a significant negative correlation between the ARHL-control Student's t map in G3 and the mean gradient scores of the control group in G3 (Pearson'r = -0.609, *p* = 0.021, 10, 000 spin permutation tests) (Supplementary Figure S3B), suggesting that regions with higher gradient scores tended to exhibit smaller ARHL-control *t* values.

### 3.4 Between-group comparisons in subcortical-cortical connectivity

**Figure 4:**
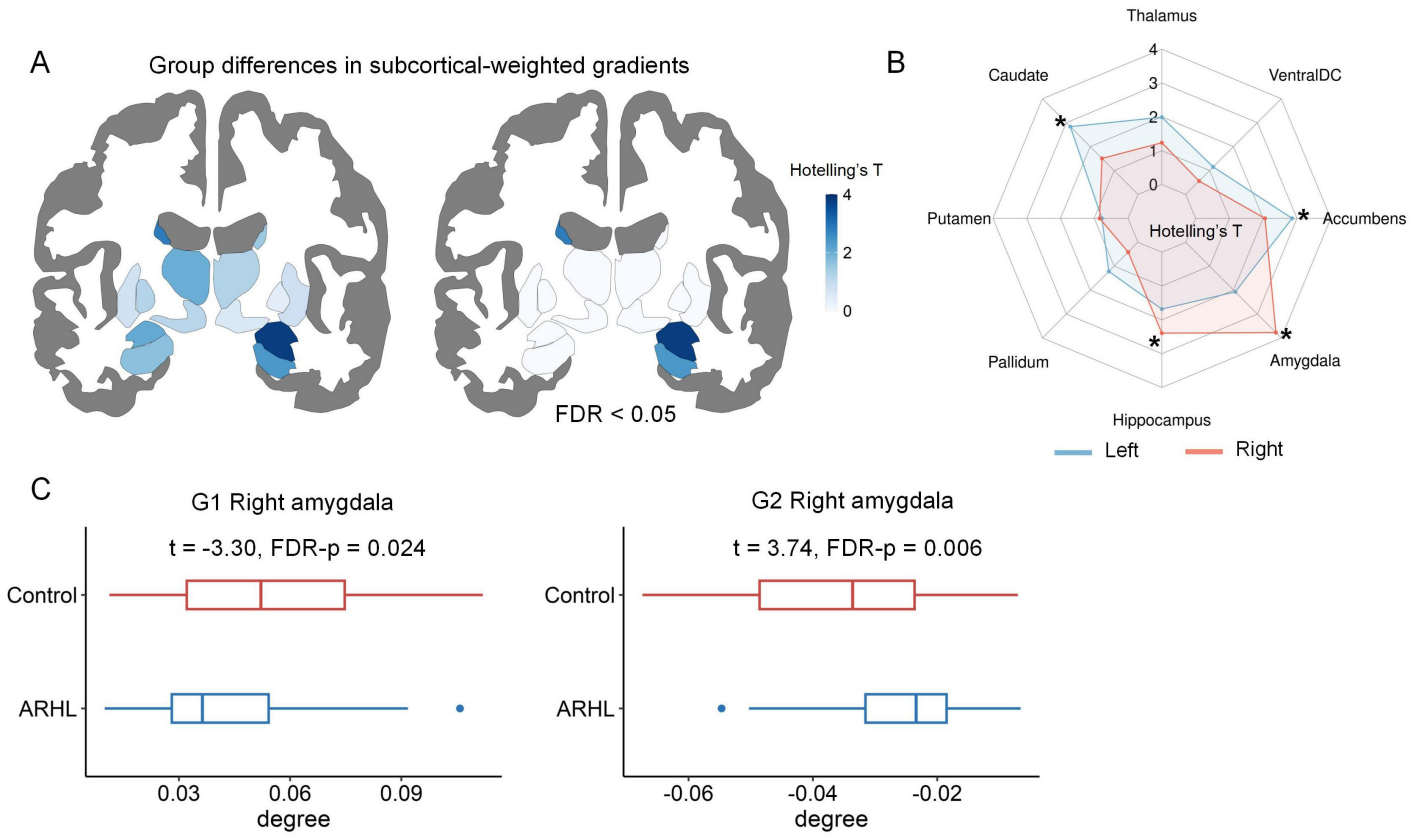
The comparisons of subcortical-weighted gradients between controls and ARHL patients. **(A)** The ARHL-control differences in the first three subcortical-weighted gradients identified by multivariate analyses. The subcortical maps are colored according to Hotelling's *T*-values. **(B)** The spider chart provides a detailed summary of Hotelling's **T** values for each subcortical region. *denotes significant group differences (FDR-corrected *p* < 0.05). **(C)** Significant group differences in the degree values of subcortical-weighted gradients in single-gradient comparisons. In each boxplot, the central line of the box indicates the median, the top/bottom edge of the box denotes the 75th/25th percentiles, and the whiskers of the box indicate the upper/lower bounds of 1.5 × the interquartile range.

Using subcortical-weighted gradients, we investigated group differences in subcortical-cortical connectivity. Multivariate analyses revealed significant ARHL-related alterations in subcortical-weighted gradients in the left caudate, left nucleus accumbens, right hippocampus, and right amygdala (Figures 4A, B). When comparisons at a single gradient, significant group differences in the degree values of subcortical-weighted gradients were detected in G1 and G2 (Figure 4C). To be specific, for G1, ARHL patients showed significantly reduced degree values in the right amygdala. For G2, ARHL patients had significantly increased degree values in the right amygdala. The details of differences in subcortical-cortical connectivity can be found in Supplementary Table S3. We provided two additional tables (Supplementary Tables S4–S5) to systematically summarize the significant results of multivariate and univariate analyses.

### 3.5 Transcriptomic signatures of altered structural gradients

**Figure 5:**
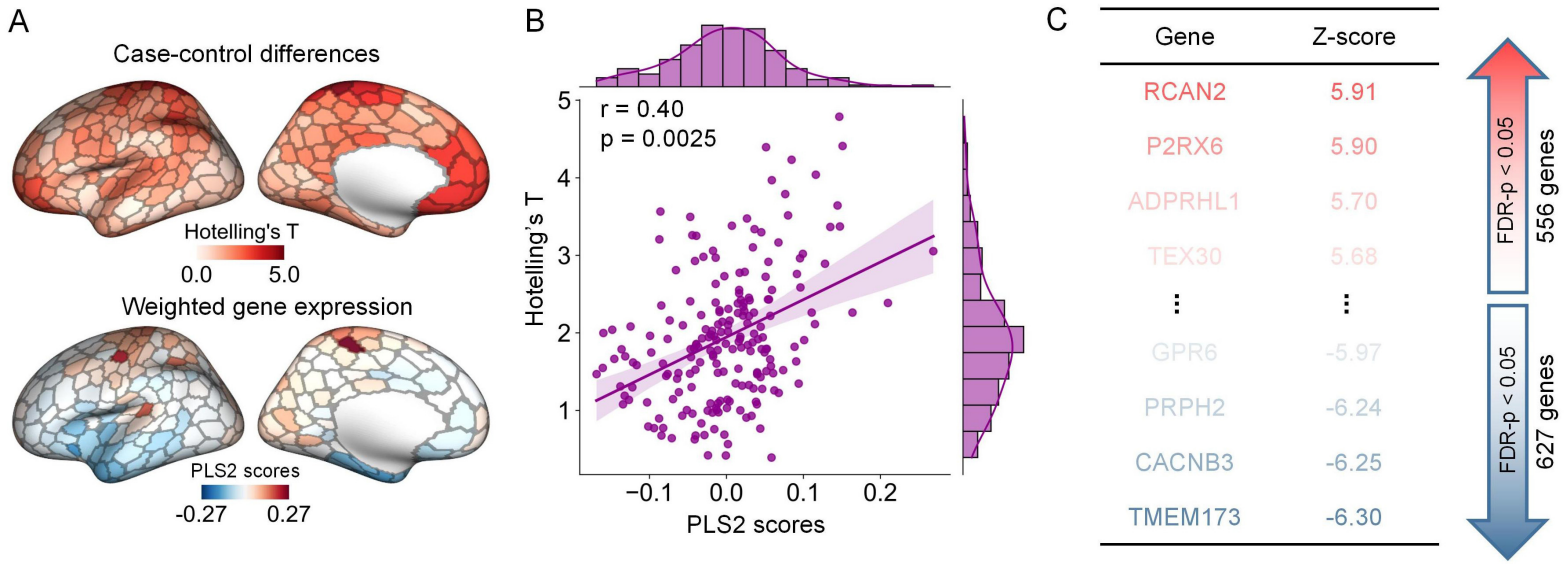
The association between alterations in structural gradients and gene expression profiles. **(A)** The graph at the top indicates the ARHL-control Hotelling's-T map of left hemisphere. The graph at the bottom indicates the weighted gene expression of left hemisphere that derived from the scores corresponding to the second component of the PLS model. **(B)** Pearson correlation between the PLS2 weighted gene expression pattern and the differences in structural gradients (*p* = 0.0025, 10,000 spin permutation tests). **(C)** Ranked PLS2 genes according to their corresponding z scores.

Using postmortem data from the AHBA database and the partial least squares (PLS) regression, we asked whether the abnormalities in structural gradients were related to gene expression profiles. We found that the PLS1 and PLS2 components accounted for 15% and 16% of the variance in group differences in gradients, significantly exceeding the null expectation (*p* = 0.0015). We focused on the PLS2 component to explore transcriptomic associations because it explained the highest variance in our PLS models. We found that the PLS2 weighted gene expression pattern was significantly correlated with the between-group differences in structural gradients. (Pearson'r = 0.40, *p* = 0.0025) (Figures 5A, B).

**Figure 6:**
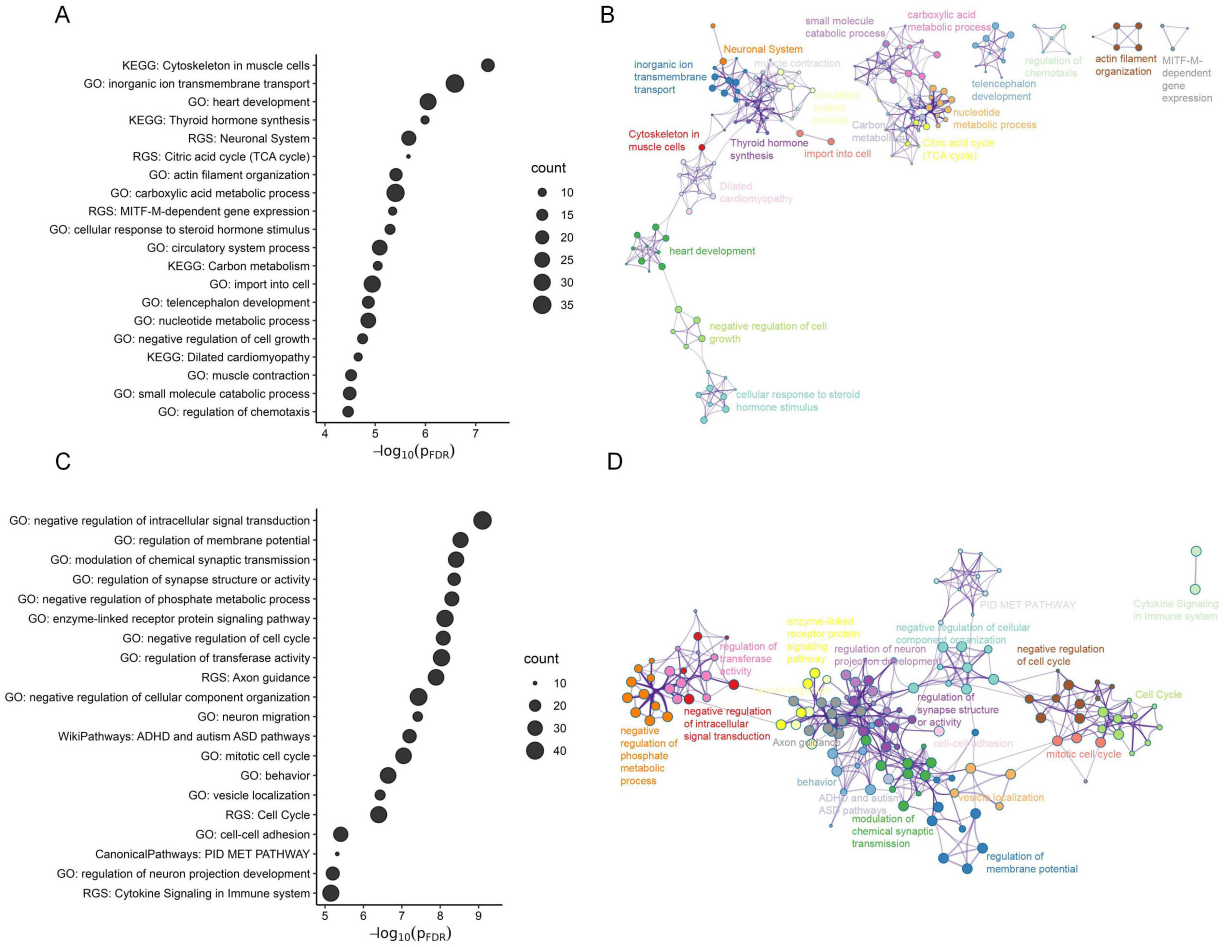
The functional enrichment of significant PLS2 genes. **(A, C)** The bubble chart for the PLS2+ **(A)** and PLS2- genes **(C)**, which shows significant GO biological processes and pathway terms (FDR-corrected *p* < 0.05). The size of the bubble reflects the number of genes contained in each term. **(B, D)** Metascape enrichment network illustrating the intra-cluster and inter-cluster similarities among enriched terms for the PLS2+ **(B)** and PLS2- **(D)** genes. Each circle point denotes an enriched term, with the point size proportional to the number of input genes included in the term. Different colors represent distinct clusters.

Utilizing the online software Metascape, we conducted gene set enrichment analyses to assess the biological significance of genes strongly contributing to the PLS2 component. The contributions of genes were defined according to their normalized weights. Based on one-sample Z tests, we extracted significant PLS2 genes including 556 PLS2+ genes (Z > 2.89, corrected p < 0.05) and 627 PLS2- genes (Z < -2.89, corrected *p* < 0.05) (Figure 5C). We chose significant enrichment terms (corrected *p* < 0.05) and removed discrete enrichment clusters. The enrichment analysis revealed that the PLS2+ genes were most prominently enriched with inorganic ion transmembrane transport (GO biological process) and Cytoskeleton in muscle cells (KEGG pathway) (Figures 6A, B). The PLS2- genes were significantly enriched for GO terms related to biological regulation and regulation of biological process, such as negative regulation of intracellular signal transduction, regulation of membrane potential, and modulation of chemical synaptic transmission (Figures 6C, D). The detailed results of enrichment analyses were shown in Supplementary Tables S6–S7.

### 3.6 Sensitivity analysis

We repeated our multivariate analyses under various methodological considerations, including (1) constructing the template gradients based on the group-average structural connectome derived exclusively from the control group; (2) calculating the affinity matrix using other similarity measures including Spearman's rank correlation and normalized angle similarity; (3) applying different thresholds to sparsify the structural connection matrix (sparsity levels including 0.7, 0.8, and 0.9); (4) using different parameter settings of diffusion map embedding ((*t*, α) = (0, 0.2), (0, 0.8), and (1, 0.5)); (5) aligning individual-level gradients with the template gradients through joint embedding (Nenning et al., 2020; Xu et al., 2020), instead of the Procrustes alignment; (6) Between-group comparisons including the average absolute motion of individual diffusion data or total outliers of individual diffusion images, derived from FSL's eddy correction process (Andersson and Sotiropoulos, 2016), as a covariate. We found that there were no significant differences in these two metrics between the control and ARHL groups (Control-ARHL, the average absolute motion: Student's t = -1.66, p-value = 0.10; total outliers: Student's t = -0.46, p-value = 0.64). The results of sensitivity analysis were shown in Supplementary Figures S4–S9. To assess the robustness, we calculated the Dice index between the significant results from the main analysis and those from the sensitivity analysis. The Dice indices for the analyses of network-level, region-level, and subcortical-weighted gradients were 0.84 ± 0.20, 0.71 ± 0.22, and 0.85 ± 0.16 (Supplementary Table S8), respectively, suggesting that our results exhibited good consistency across various methodological considerations.

We validated our findings using different brain atlas (Glasser atlas) (Glasser et al., 2016). We found that the verification results were highly consistent with the main results (Supplementary Figure S10). We also added an alternate parcellation that derived from von Economo and Koskina's cytoarchitectonic stratification (von Economo and Koskinas, 1925; Larivière et al., 2021), which grouped 400 regions into five distinct structural types: agranular, frontal, parietal, polar, and granular. We found that ARHL patients showed significantly abnormal structural gradients in three structural types including agranular, frontal, and polar (Supplementary Figure S11).

Considering that there was an imbalance in the sample size between the control (28 participants) and ARHL (49 participants) groups. We conducted optimal group matching using the R package MatchIt (Ho et al., 2011), with group as the treatment variable and age and sex as covariates. This process yielded a subset including 28 controls and 28 ARHL patients. We repeated our main analysis in this subset. The Dice indices for the network-level, region-level, and subcortical-weighted gradient analyses were 0.74, 0.99, and 1, respectively, indicating good reproducibility (Supplementary Figure S12, Supplementary Table S8).

## 4 Discussion

In the current study, we employed the gradient mapping framework to investigate the hierarchical organization of structural connectome in ARHL patients. We found that ARHL patients showed disruptions of connectome organization in multiple functional networks, including the somatomotor, dorsal attention, limbic, and default mode networks. Multivariate analyses at the regional level revealed atypical structural gradients mainly in the lateral parietal cortex, lateral temporal cortex, cingulate, right visual, somatomotor, and orbitofrontal cortex. Univariate analyses further indicated that these alterations in structural gradients were concentrated in the third gradient, with ARHL patients showing significantly decreased gradient scores in the somatomotor and superior parietal areas. Using subcortical-weighted gradients, we observed significant between-group differences in subcortical-cortical connectivity in the left caudate, left nucleus accumbens, right hippocampus, and right amygdala. Transcriptomic association analyses suggested that these alterations in structural gradients related to weighted gene expression profiles, with strongly contributing genes primarily enriched for the “Cytoskeleton in muscle cells” pathway, inorganic ion transmembrane transport, and regulation of biological process. In sum, these findings provide evidence of structural gradient reorganization in ARHL patients and uncover potential molecular underpinnings behind these changes.

Prior research has demonstrated that ARHL patients exhibit significantly aberrant white matter integrity (e.g., decreased fractional anisotropy) (Ma et al., 2016), altered structural connectivity strength, and subtle differences in graph metrics (e.g., global efficiency) of structural connectomes (Ponticorvo et al., 2022). Here we extend these diffusion MRI findings in ARHL using a gradient mapping method that compresses high-dimensional structural connectomes into a range of low-dimensional continuous representations in the embedded space. Prior studies employing a similar methodology have reported abnormal hierarchy of the macroscale structural connectome in somatomotor and association cortices in patients with autism (Park et al., 2021b), as well as disrupted structural connectome organization in the sensory and limbic regions in patients with episodic migraine (Noh et al., 2024). By multivariate analyses at the network and region levels, we found that patients showed broad and distributed structural gradient abnormalities across cortical regions, spanning from the primary sensory cortex (e.g., somatomotor and right visual regions) to the high-order cognitive cortex (e.g., orbitofrontal and inferior parietal regions). Structural gradient reorganizations observed in the primary sensory regions may arise from the primary sensory cortex's compensation in response to impaired auditory input (Glick and Sharma, 2017; Tong et al., 2023). In contrast, structural gradient abnormalities in the high-order association regions are likely associated with cognitive deficits (Slade et al., 2020). In addition, our findings are in agreement with the emerging evidence that ARHL involves disruption of multi-network systems (Chen et al., 2018; Tong et al., 2023; Xing et al., 2022). Post-hoc annlyses and univariate analyses at the single-gradient level suggested that the overall effects of ARHL on structural gradients were primarily contributed by G3. In this study, G3 represents a spatial axis where the highest gradient values are in the parietal/motor regions while the lowest values are in the prefrontal regions. Interestingly, we observed significantly reduced gradient values in the somatomotor and parietal regions, along with increased values in the left prefrontal regions in ARHL patients, suggesting a compressed hierarchical organization. The networks or regions with significant differences that we identified overlapped to some extent with previous fMRI-based studies in ARHL patients. For example, prior studies have reported altered functional connectivity within the default mode network (Xing et al., 2022) and abnormal functional organizations of the visual network in ARHL patients (Ponticorvo et al., 2022). One recent study applied the diffusion mapping embedding method to functional connectome and found significantly altered functional gradients in the visual, default mode, somatomotor, frontoparietal, and limbic networks (Tong et al., 2023). Our findings provide a potential structural substrate for the widespread functional connectome abnormalities reported in ARHL patients. Further studies are required to integrate functional and diffusion data to dissect how structural connectome reorganizations affect functional connectome in ARHL patients. By meta-analysis, we observed that regions with significant differences were mainly related to motion-related cognitive terms such as motor imagery and premotor. Previous studies have suggested a decrease in auditory-motor processing of speech for ARHL patients, indicating a reduced integration of the motor cortex during phonological processing (Panouillères and Möttönen, 2018). However, due to the lack of detailed levels of motor function in patients, the direct link between altered structural gradients and motor function warrants further investigation.

Through multivariate and univariate analyses at both the network-level and region-level, we observed that the large effect sizes and great statistical significance for between-group differences in structural gradients were located in the somatomotor regions, as well as the superior parietal lobule. Previous studies indicate that these regions might be implicated in the pathology of ARHL. For instance, ARHL patients exhibit altered functional gradients in the somatomotor network (Tong et al., 2023), and increased betweenness centrality of the functional network in the right postcentral gyrus (Guan et al., 2022). Furthermore, ARHL can affect resting-state functional connectivity between the dorsal attention network and superior parietal lobule (Rosemann and Thiel, 2019). A previous study has suggested that the somatomotor system acts as a transdiagnostic hub that is associated with cognitive dysfunction, general psychopathology, and impulsivity (Kebets et al., 2019). Our findings offer further support for the pathological relevance of the somatomotor and superior parietal regions, implying that targeting these regions potentially contributes to the diagnosis and treatment of ARHL.

The alterations of structural connectome hierarchy observed in ARHL patients indicate the abnormality of the white matter structure. which is compatible with previously reported impairments of white matter integrity in ARHL patients (Ma et al., 2016). It's worth noting that the abnormalities of white matter integrity are found in the hearing-related brain regions. Similarly, we observed altered structural gradients in the left superior temporal lobe region near the auditory cortex. Furthermore, in contrast to local white matter integrity abnormalities, we observed extensive structural gradient alterations ranging from sensory to association regions. We speculate that the long-term impairments in local sensory inputs propagate and affect multiple functional systems (e.g. the somatomotor and default mode networks), ultimately disrupting higher-order cognitive functions. Prior studies on autism and schizophrenia have observed a comparable cascading effect, where anomalies in the sensory system impact higher cognitive systems (Park et al., 2021b; Dong et al., 2023). A recent study suggests that children with congenital sensorineural hearing loss exhibit a functional reorganization involving the auditory, somatic motor, visual, and prefrontal cortices (Yin et al., 2024). This is consistent with our findings, implying that abnormal auditory function can lead to widespread alterations across multiple systems. Based on the gradient mapping framework, previous work has demonstrated that the aging process (Wang et al., 2024b) and neurodegenerative diseases such as Alzheimer's disease (Wang et al., 2024a) and frontotemporal dementia (Bouzigues et al., 2024) exhibit altered hierarchical organization of functional connectome in specific networks such as the default mode, somatomotor, and ventral attention networks. Although these reported networks partially overlap with our findings on ARHL, these studies are based on the functional connectome. Future research should further investigate whether and how other atypical age-related processes change the hierarchical organization of structural connectome, to determine if there is an overlap with our findings.

Several studies have documented notable changes in subcortical structures of ARHL patients, including atrophy of the hippocampus and amygdala (Belkhiria et al., 2020; Jafari et al., 2021), increased functional connectivity between the caudate and right supramarginal gyrus (Xu et al., 2022), and reduced directed functional connectivity between the hippocampus and cortical areas (Chen et al., 2020). By employing subcortical-weighted gradients, we observed altered subcortical-cortical structural connectivity in the left caudate, left nucleus accumbens, right hippocampus, and right amygdala. Our finding, combined with previous findings, provides emerging evidence that these subcortical regions are intricately related to the pathology of ARHL. These subcortical structures are generally involved in motor processes, memory, and learning, partially mirroring the cognitive functions of cortical areas we have identified. This finding further suggested that ARHL patients likely exhibit impairments that extend from the primary motor to higher-order cognitive systems. Multivariate and single-gradient comparisons consistently demonstrated that the right amygdala had the strongest effect of group differences. Given the crucial role of the amygdala in emotional response and social cognition (Phelps, 2006), we speculate that abnormal subcortical-cortical connectivity in the right amygdala might stem from social isolation and loneliness caused by long-term hearing loss (Husain et al., 2014). Collectively, our findings highlight that subcortical regions, particularly the amygdala, are crucial for understanding the pathological mechanism behind ARHL.

By utilizing transcriptome data from the AHBA database, we identified a link between ARHL-related changes in structural gradients and weighted gene expression profiles. The enrichment analysis informed that the most correlated genes were primarily enriched for the ‘Cytoskeleton in muscle cells' pathway and several biological processes including inorganic ion transmembrane transport (GO:0098660) and terms related to regulation of biological process. These terms that we identified exhibit some correspondences with molecular mechanisms associated with ARHL as reported in the prior literature. More specifically, prior studies have suggested that specific ion channels and transport proteins (e.g., KCNQ4 K+ channel) are crucial for normal hearing (Bazard et al., 2021). The aging process disrupts protein homeostasis in the inner ear, resulting in alterations in ionic homeostasis that induce ARHL-related dysfunction (Peixoto Pinheiro et al., 2021; Guo et al., 2022). Animal experiments also indicate that the regulation of specific membrane potentials (e.g., mitochondrial membrane potential) may be associated with ARHL (Tian et al., 2020; Guo et al., 2022). Our imaging-transcriptomics findings contribute to the understanding of the molecular substrates underlying ARHL.

There are a few limitations to our findings that warrant consideration. First, the relatively modest sample size in the current study potentially constrains the generalizability of our findings. Future studies could utilize a large sample to validate our findings. Second, due to the dearth of detailed information on the severity of hearing loss, and cognitive status and behaviors, we are unable to explore the impacts of these factors on the structural gradient differences. Further research aimed at investigating whether structural connectome reorganization relates to these factors would be of great significance. Third, our findings are based on single-modal neuroimaging data. Future studies could integrate other data types (e.g., functional MRI, magnetic resonance spectroscopy, or longitudinal data) to further validate and expand our findings. Finally, our transcriptomic association analyses relied on gene expression data obtained from donors without ARHL. Future studies should leverage transcriptomic data from ARHL patients to further validate our findings.

## Data Availability

The original contributions presented in the study are included in the article/Supplementary material, further inquiries can be directed to the corresponding authors.
