# Supplementary material for: Disruption of structural connectome hierarchy in age-related hearing loss
Source: Front Neurosci. 2025 Mar 17;19:1555553. doi: 10.3389/fnins.2025.1555553 (PMC11955685; doi:10.3389/fnins.2025.1555553)
Supplement: Supplementary file 2 [file Data_Sheet_1.pdf]

Supplementary Material

1 SUPPLEMENTARY FIGURES

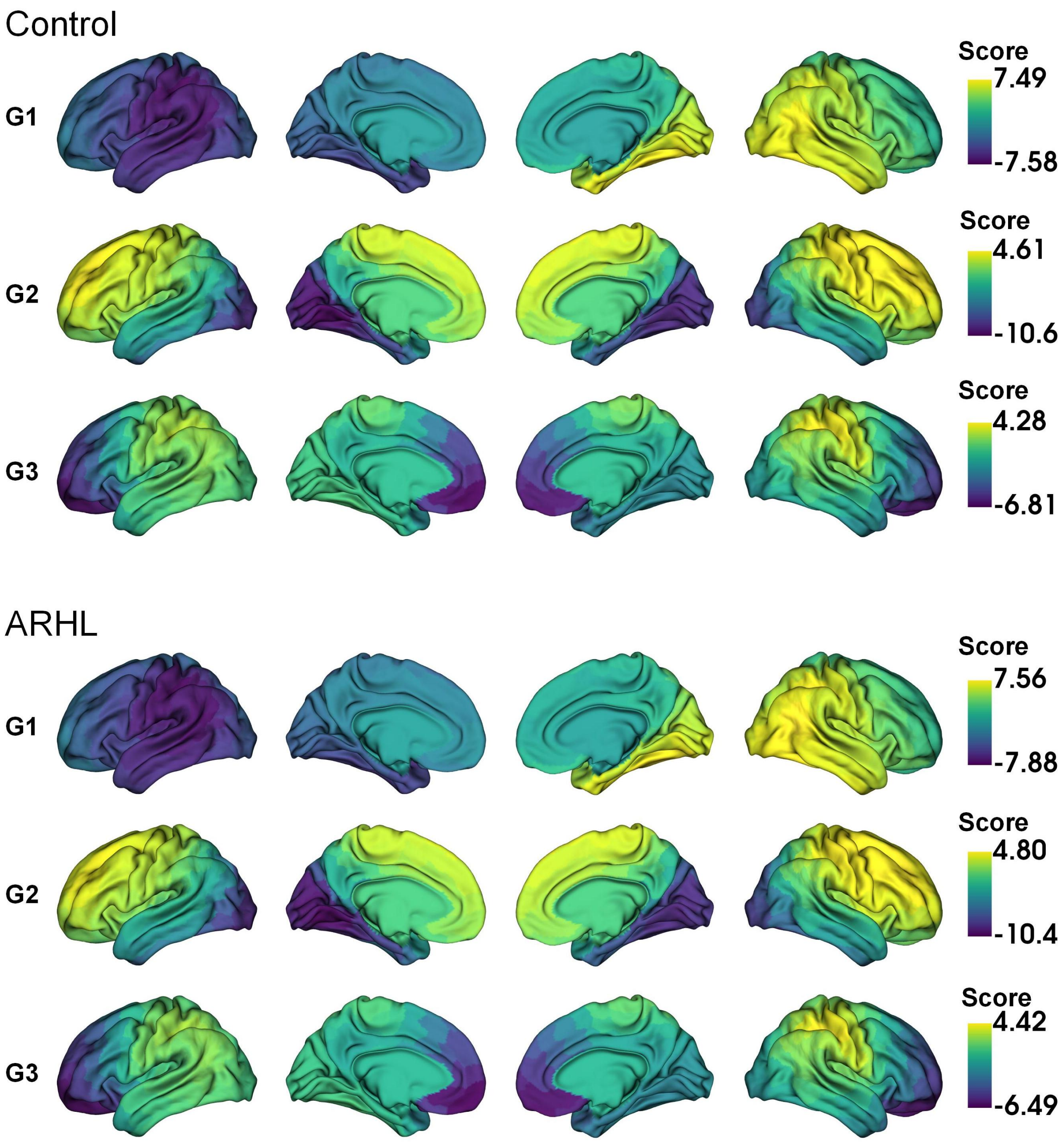

Figure S1. The first three structural gradients of the group averages for the control and ARHL groups.

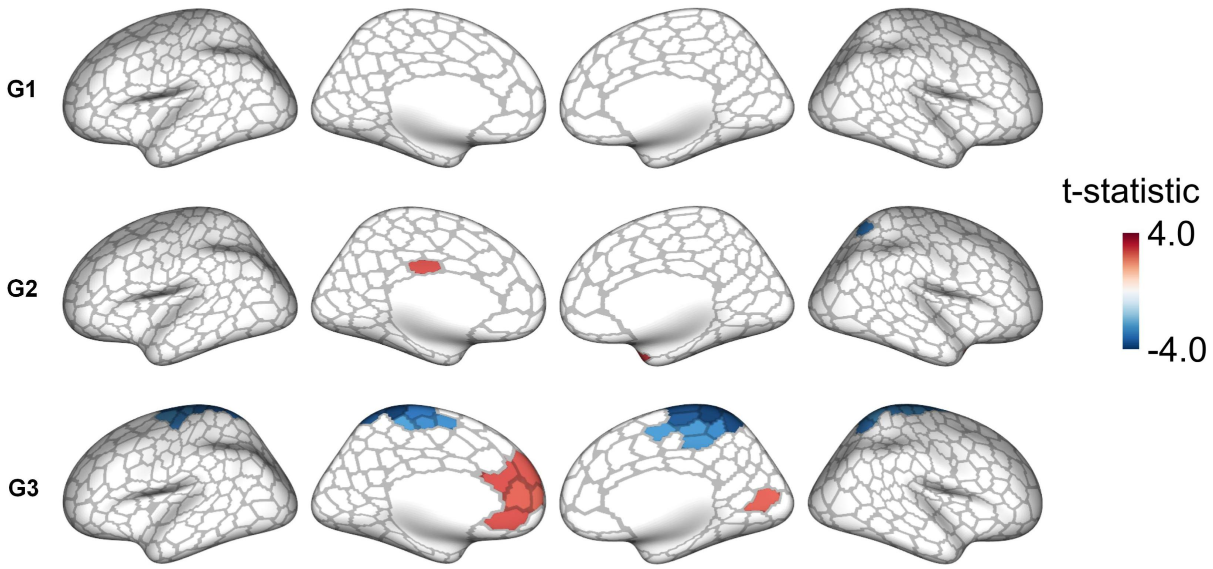

Figure S2. Post-hoc ARHL-control differences at the single-gradient level. Significant ARHL-control differences are seen only for G2 and G3 ($p < 0.05/3$).

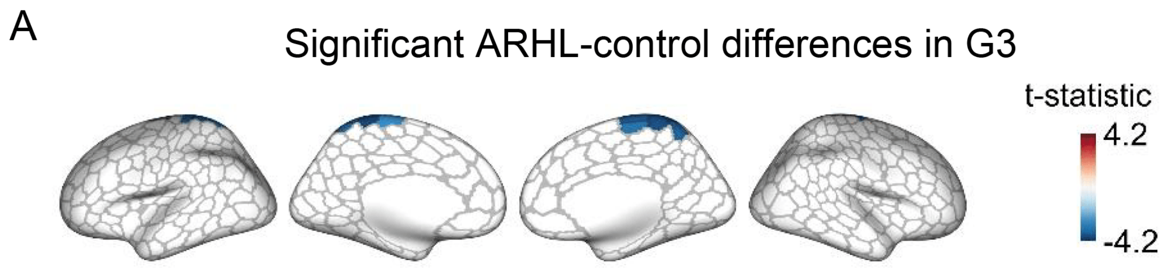

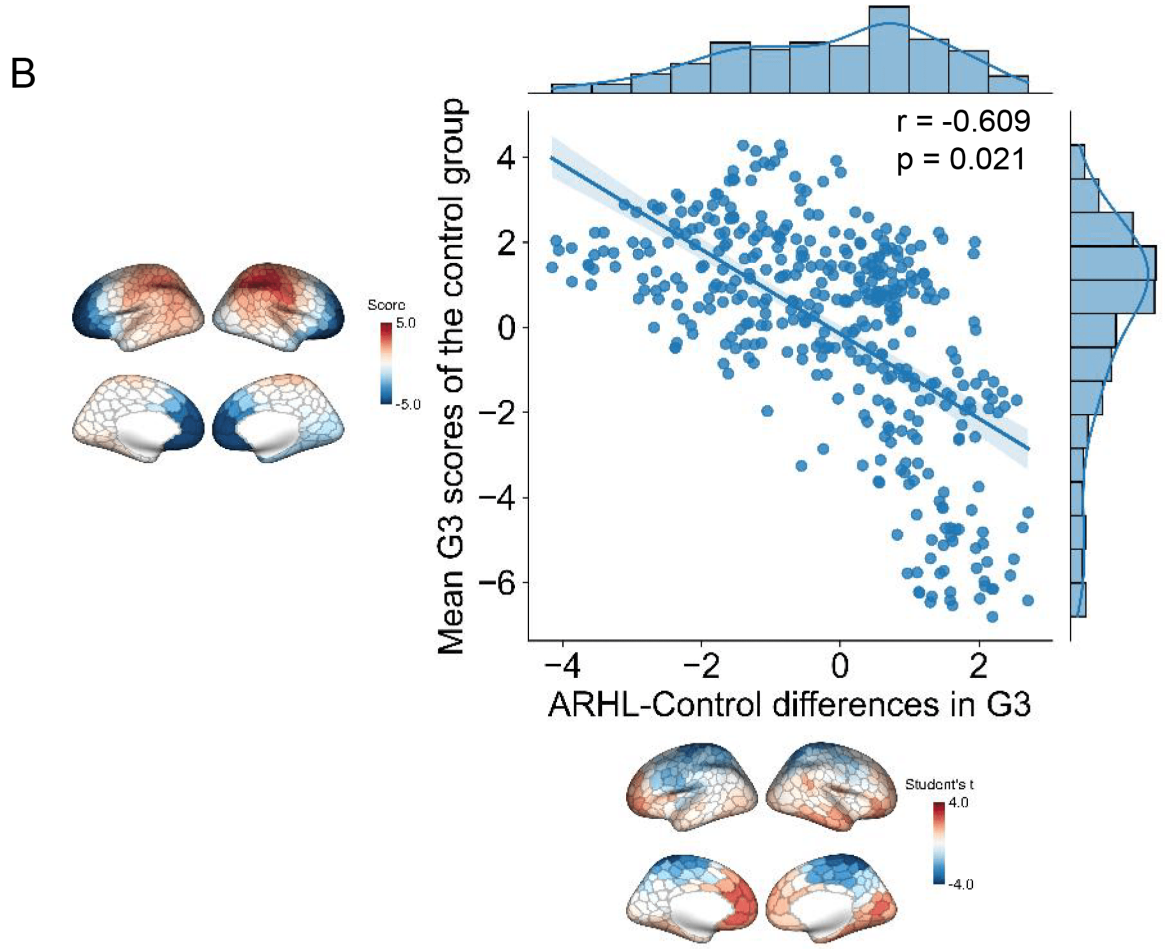

Figure S3. ARHL-control differences in G3 using the univariate linear model. A. Significant ARHL-control differences in G3 (FDR-p < 0.05). B. The spatial correlation between the ARHL-control t map and mean G3 scores of the control group (Pearson's r = -0.609, p-value = 0.021, 10, 000 spin permutation tests).

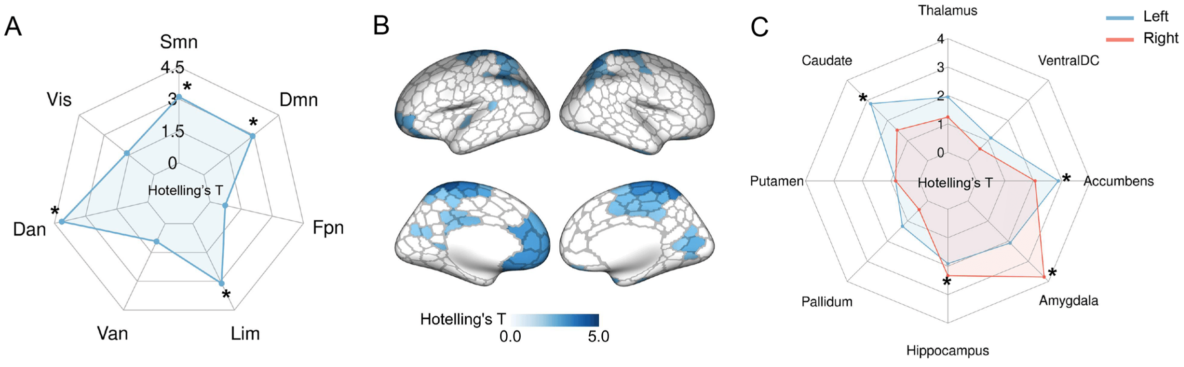

Figure S4. We constructed the template gradients based on the group-average structural connectome derived only from the control group and repeated our main analyses. A. Network-level comparisons of structural gradients between controls and ARHL patients using multivariate analyses. B. Significant ARHL-control differences in the first three structural gradients using multivariate analyses in region-level comparisons. C. The ARHL-control differences in the first three subcortical-weighted gradients using multivariate analyses. $*$ denotes significant group differences.

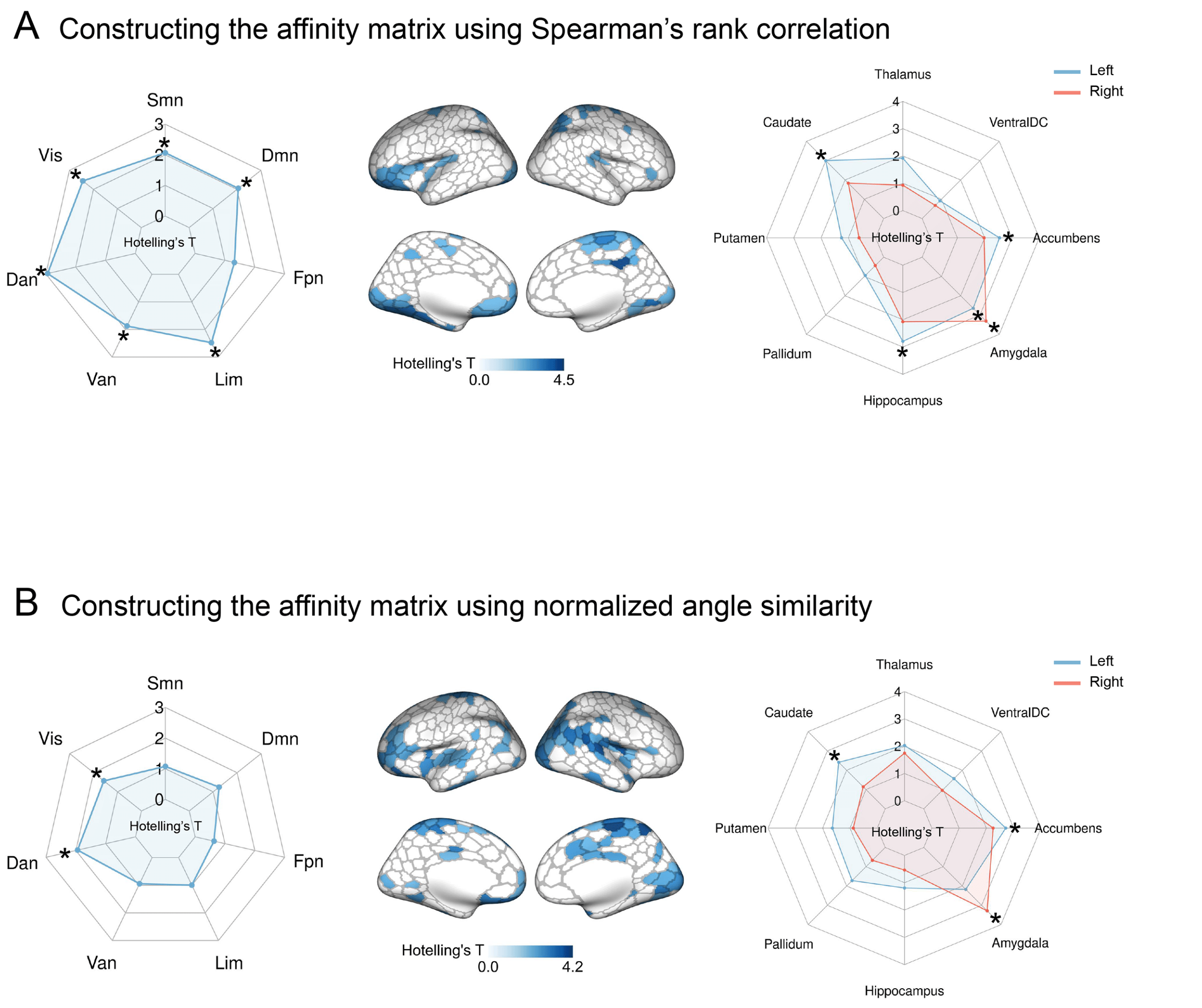

Figure S5. We calculated the affinity matrix using two different similarity measures and repeated our multivariate analyses. A. The affinity matrix was calculated using Spearman's rank correlation. B. The affinity matrix was calculated using normalized angle similarity. For A & B, the graph on the left indicated network-level comparisons of structural gradients between controls and ARHL patients. The graph in the middle showed significant ARHL-control differences in the first three structural gradients in region-level comparisons. The graph on the right displayed the ARHL-control differences in the first three subcortical-weighted gradients. $*$ denotes significant group differences.

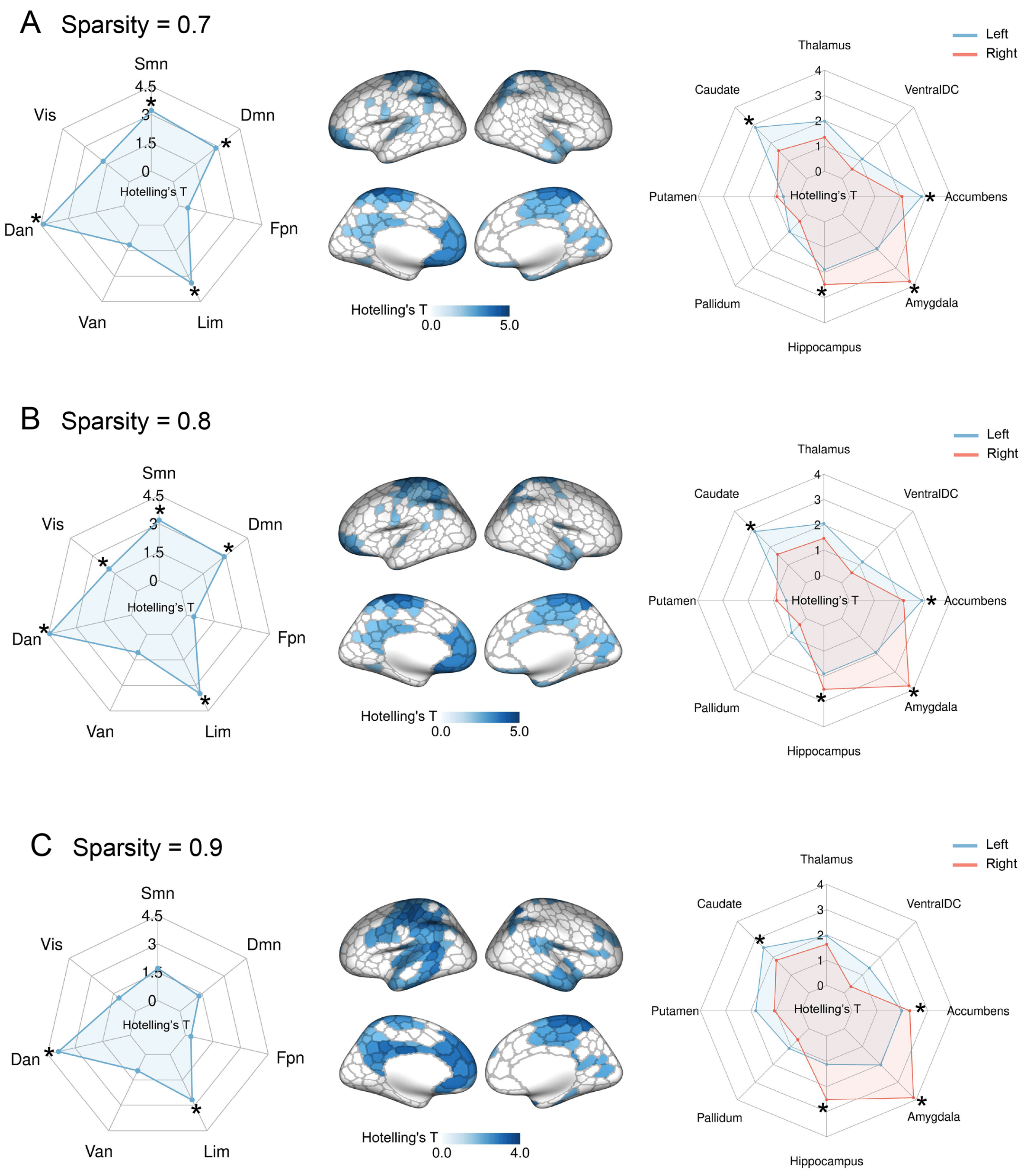

Figure S6. We applied different thresholds to sparsity the structural connectome and repeated our multivariate analyses. A. Sparsity level = 0.7. B. Sparsity level = 0.8. C. Sparsity level = 0.9. For A, B, and C, the graph on the left indicated network-level comparisons of structural gradients between controls and ARHL patients. The graph in the middle showed significant ARHL-control differences in the first three structural gradients in region-level comparisons. The graph on the right displayed the ARHL-control differences in the first three subcortical-weighted gradients. $*$ denotes significant group differences.

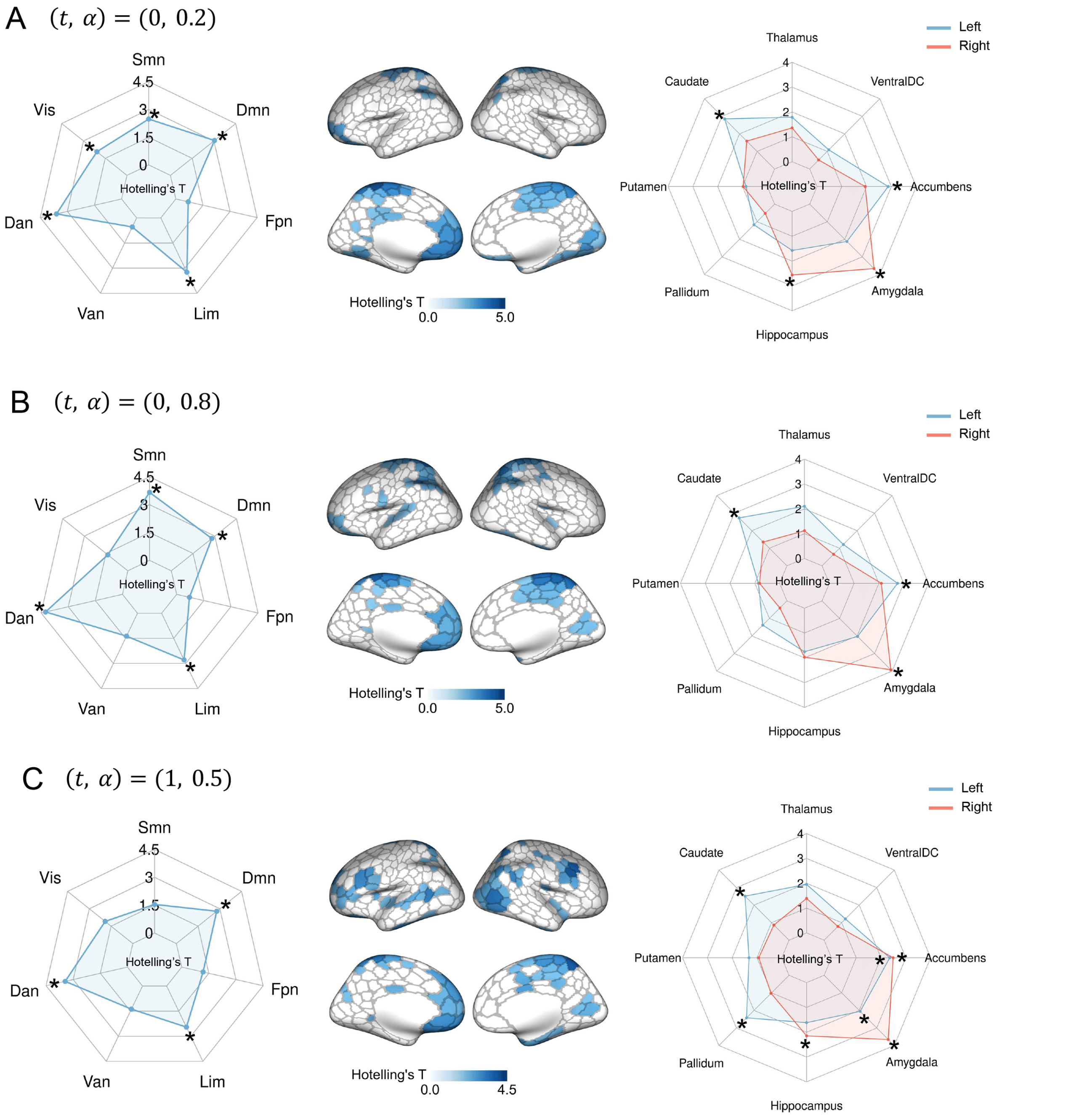

Figure S7. We used different parameter settings of diffusion map embedding and repeated our multivariate analyses. A. $(t, \alpha) = (0, 0.2)$. B. $(t, \alpha) = (0, 0.8)$. C. $(t, \alpha) = (1, 0.5)$. For A, B, and C, the graph on the left indicated network-level comparisons of structural gradients between controls and ARHL patients. The graph in the middle showed significant ARHL-control differences in the first three structural gradients in region-level comparisons. The graph on the right displayed the ARHL-control differences in the first three subcortical-weighted gradients. $*$ denotes significant group differences.

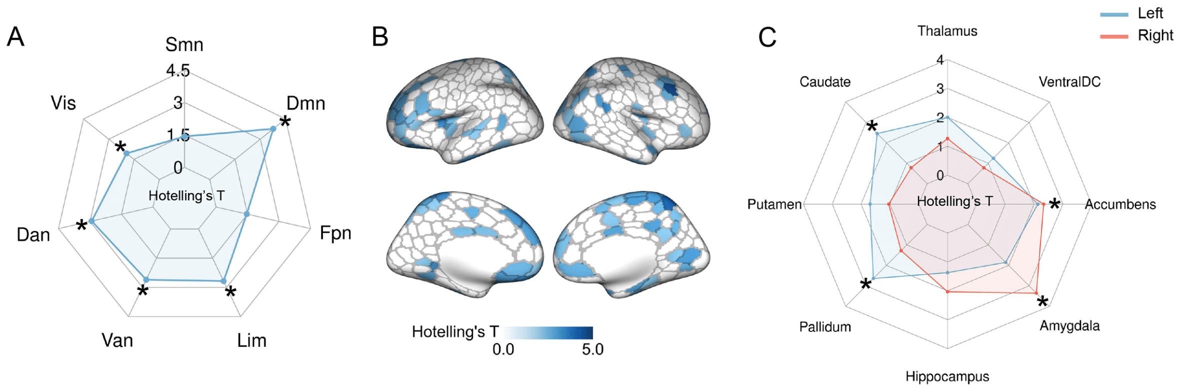

Figure S8. We aligned individual-level gradients with the template gradients through joint embedding and repeated our multivariate analyses. A. Network-level comparisons of structural gradients between controls and ARHL patients. B. Significant ARHL-control differences in the first three structural gradients in region-level comparisons. C. The ARHL-control differences in the first three subcortical-weighted gradients. $*$ denotes significant group differences.

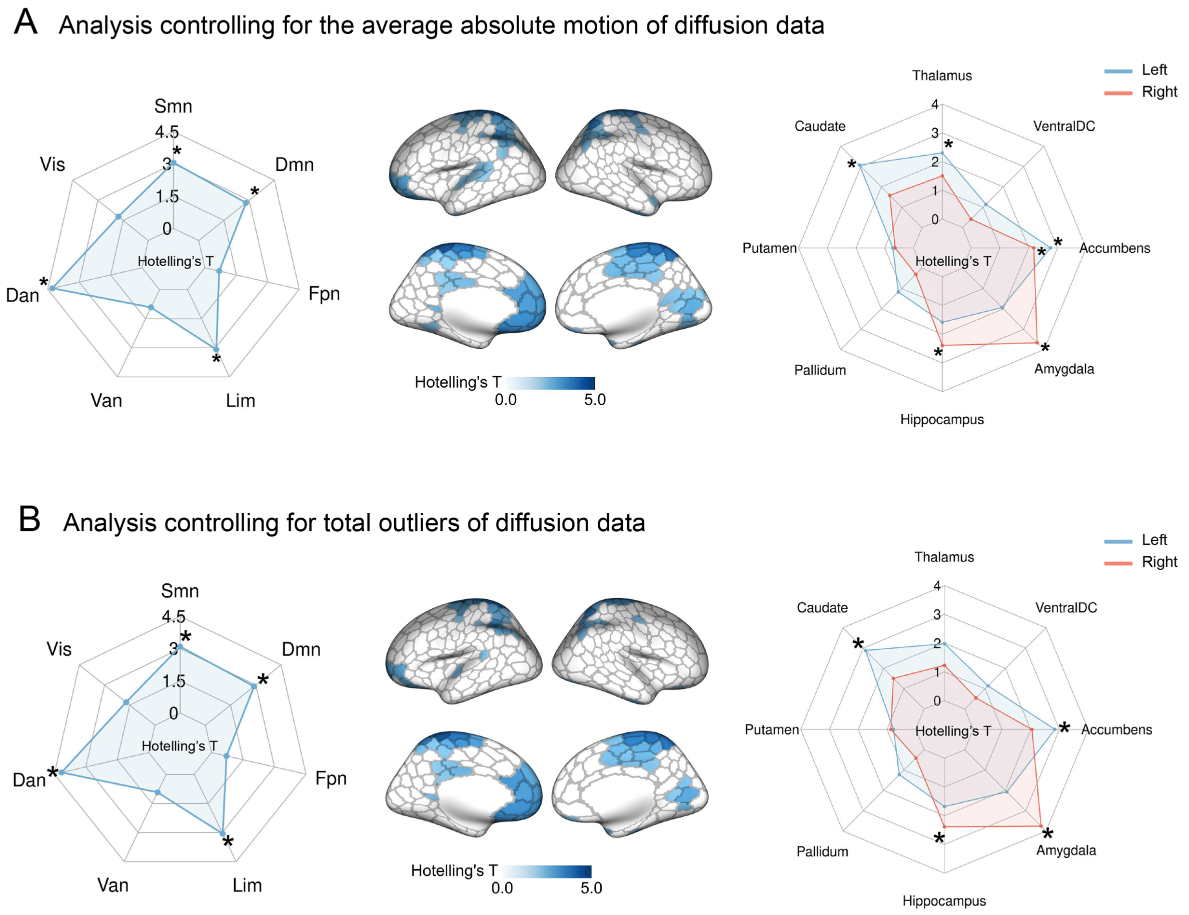

Figure S9. We included some covariates reflecting image quality and repeated multivariate analyses. A. Multivariate analyses controlling for the average absolute motion of diffusion data. B. Multivariate analyses controlling for total outliers of diffusion data. For A & B, the graph on the left indicated network-level comparisons of structural gradients between controls and ARHL patients. The graph in the middle showed significant ARHL-control differences in the first three structural gradients in region-level comparisons. The graph on the right displayed the ARHL-control differences in the first three subcortical-weighted gradients. $*$ denotes significant group differences.

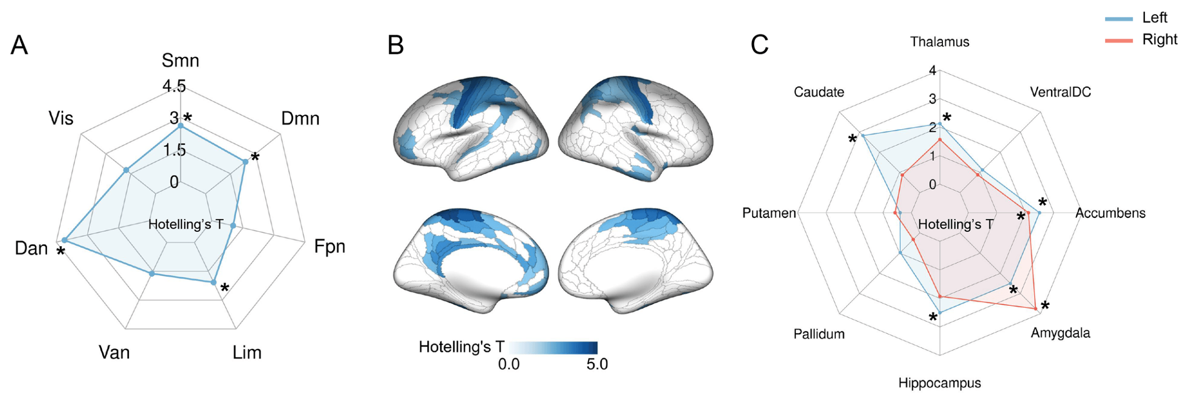

Figure S10. We constructed individual structural connectome using the Glasser atlas and repeated our main analyses. A. Network-level comparisons of structural gradients between controls and ARHL patients using multivariate analyses. B. Significant ARHL-control differences in the first three structural gradients identified by multivariate analyses. C. The ARHL-control differences in the first three subcortical-weighted gradients using multivariate analyses. ∗ denotes significant group differences.

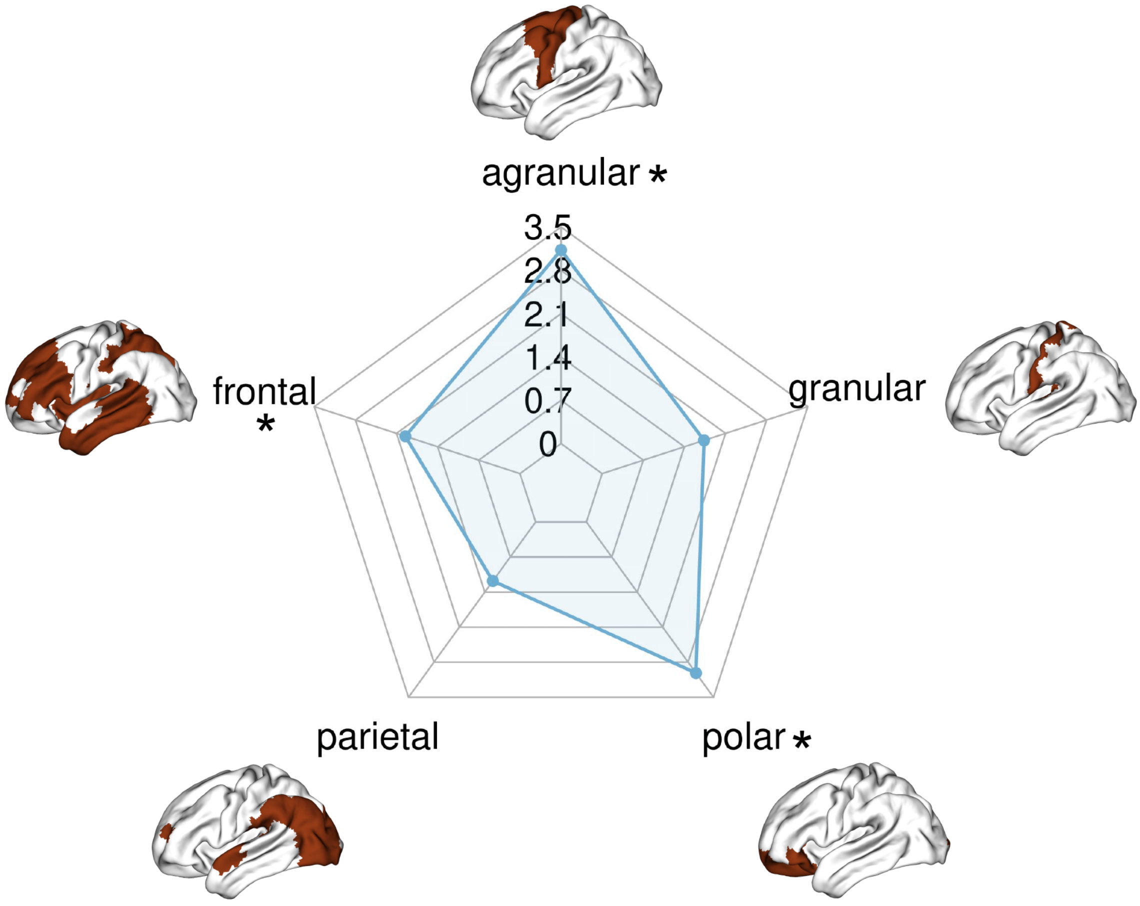

Figure S11. We grouped 400 functional parcels based on von Economo and Koskina's cytoarchitectonic stratification. We observed that ARHL patients showed significantly abnormal structural gradients in three structural types including agranular, frontal, and polar. ∗ denotes significant group differences.

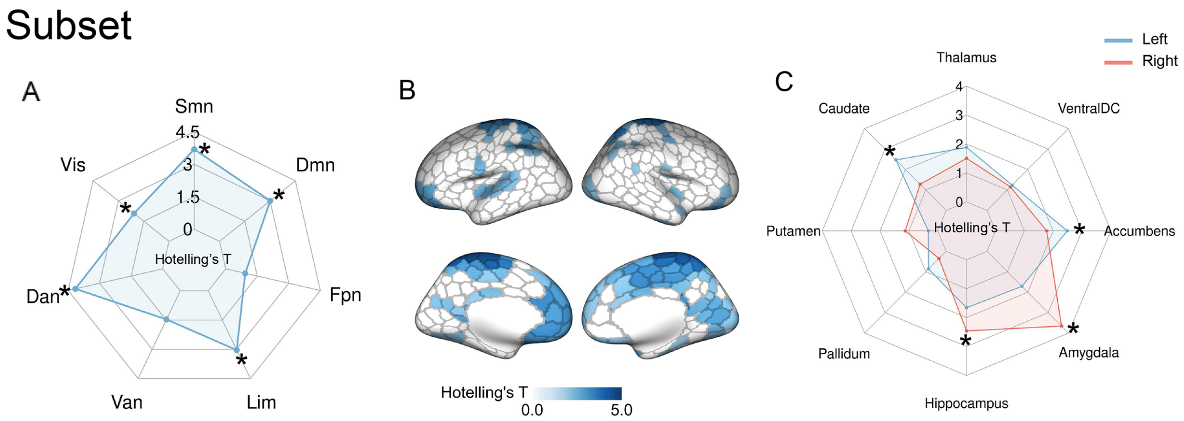

Figure S12. We validated our findings in a subset, which was obtained by optimal group matching. A. Network-level comparisons of structural gradients between controls and ARHL patients using multivariate analyses. B. Significant ARHL-control differences in the first three structural gradients identified by multivariate analyses. C. The ARHL-control differences in the first three subcortical-weighted gradients using multivariate analyses. $*$ denotes significant group differences.
